# Transcript profiles uncover temporal and stress-induced changes of metabolic pathways in germinating sugar beet seeds

**DOI:** 10.1186/1471-2229-8-122

**Published:** 2008-12-01

**Authors:** Elena Pestsova, Juliane Meinhard, Andreas Menze, Uwe Fischer, Andrea Windhövel, Peter Westhoff

**Affiliations:** 1 Institut für Entwicklungs- und Molekularbiologie der Pflanzen, Heinrich-Heine-Universität, Universitätsstr. 1, 40225 Düsseldorf, Germany; 2 KWS SAAT AG, Grimsehlstr. 31, 37555 Einbeck, Germany

## Abstract

**Background:**

With a cultivation area of 1.75 Mio ha and sugar yield of 16.7 Mio tons in 2006, sugar beet is a crop of great economic importance in Europe. The productivity of sugar beet is determined significantly by seed vigour and field emergence potential; however, little is known about the molecular mechanisms underlying these traits. Both traits exhibit large variations within sugar beet germplasm that have been difficult to ascribe to either environmental or genetic causes. Among potential targets for trait improvement, an enhancement of stress tolerance is considered because of the high negative influence of environmental stresses on trait parameters. Extending our knowledge of genetic and molecular determinants of sugar beet germination, stress response and adaptation mechanisms would facilitate the detection of new targets for breeding crop with an enhanced field emergence potential.

**Results:**

To gain insight into the sugar beet germination we initiated an analysis of gene expression in a well emerging sugar beet hybrid showing high germination potential under various environmental conditions. A total of 2,784 ESTs representing 2,251 'unigenes' was generated from dry mature and germinating seeds. Analysis of the temporal expression of these genes during germination under non-stress conditions uncovered drastic transcriptional changes accompanying a shift from quiescent to metabolically active stages of the plant life cycle. Assay of germination under stressful conditions revealed 157 genes showing significantly different expression patterns in response to stress. As deduced from transcriptome data, stress adaptation mechanisms included an alteration in reserve mobilization pathways, an accumulation of the osmoprotectant glycine betaine, late embryogenesis abundant proteins and detoxification enzymes. The observed transcriptional changes are supposed to be regulated by ABA-dependent signal transduction pathway.

**Conclusion:**

This study provides an important step toward the understanding of main events and metabolic pathways during germination in sugar beet. The reported alterations of gene expression in response to stress shed light on sugar beet stress adaptation mechanisms. Some of the identified stress-responsive genes provide a new potential source for improvement of sugar beet stress tolerance during germination and field emergence.

## Background

Sugar beet (*Beta vulgaris *L.) is a major crop for sugar production in countries with a temperate climate. The agronomic productivity of sugar beet, as a direct seeded crop, is determined significantly by the uniformity of seedling emergence in the field [1]. Despite the economic importance of field emergence and a strong desire of growers to improve it, little is known about the molecular mechanisms underlying this trait, mainly due to its genetic complexity and the large environmental effects.

In the current study we concentrated our efforts on the molecular analysis of seed germination, the first stage of field emergence. Germination *sensu stricto *commences with the uptake of water by the dry seed – imbibition – and is completed when a part of the embryo, usually the radicle, extends to penetrate the structures that surround it [2]. Seed germination has been intensively investigated on the molecular level in species having oil-storing seeds, e.g. *Arabidopsis *and *Brassica *[3-7], starch-storing seeds, e.g. cereals [8,9], and protein-accumulating seeds, e.g. legumes [10,11]. Despite some interspecies differences, regulation of germination is mostly determined by the interaction between the two plant hormones, gibberellins (GAs) and abscisic acid (ABA). Whereas ABA plays a primary regulatory role in seed maturation and dormancy, GAs are essential for the induction of germination. Other plant hormones, especially ethylene and brassinosteroids (BRs), are also involved in the regulation of germination too. The presence of an extensive cross talk between the plant hormone signalling systems is suggested [12-15].

Sugar beet belongs to the *Amaranthaceae *and utilizes the starchy perisperm, a diploid maternal tissue originated from the nucellus, as a storage organ of the seed. At seed maturity the perisperm represents a dead tissue surrounded by an embryo, so that only one ('the inner') of the two cotyledons is directly adjacent to it. The 'botanically true' seed consisting of embryo and perisperm covered by a testa, is contained within a thick fruit structure called pericarp [16,17]. In addition to the starchy perisperm, which composes around 35% of the seed dry weight, sugar beet embryos accumulate also proteins (16%) and lipids (16%) as reserves [1]. The unique morphology of the sugar beet seed and the specific proportion of seed storage compounds raise the question, whether germination in sugar beet is similar to the one in the previously investigated species or follows its own program.

Only few studies have investigated sugar beet germination [1,16-21]. Recent analysis of hormone signalling during sugar beet germination [17] demonstrated that some specific features in the regulation do exist. In contrast to other species, the radicle emergence of sugar beet fruits or seeds is not appreciably affected by a treatment with GAs, BRs, auxins, cytokinins and jasmonates, but is promoted by ethylene or the ethylene precursor 1-aminocyclopropane-1-carboxylic acid (ACC). Other publications address the sugar beet seed storage reserves. Their mobilization attracted much attention because the reserves are the only source of energy for seedling growth until establishment of the photosynthetic apparatus. Elamrani et al. [1,18] showed that during early growth, sugar beet cotyledons behaved mainly as a lipid mobilization and gluconeogenic tissue thus providing substrates to the seedling. A subsequent study of de los Reyes et al. [20] supported the importance of glyoxylate cycle enzymes, which are involved in lipid catabolism, during germination under stress conditions, and suggested, for the fist time, to use these enzymes as biochemical targets for enhanced germination and improved emergence in sugar beet.

Among other potential targets for trait improvement are genes involved in the onset of seed and seedling stress tolerance [21]. Both seed germination and early seedling growth are very sensitive to biotic and abiotic stresses [22,23]. The inability of the seedling to tolerate adverse environmental conditions may result in severe damage and decreased overall field emergence. McGrath et al. [21] recently showed that sugar beet germplasm differed in germination under salt stress and reported that selection for enhanced stress tolerance during germination appeared to be feasible, since gains were observed in progeny of salt germinated seedlings. Extending our knowledge of genetic and molecular determinants of sugar beet seed germination would facilitate the detection of new targets for breeding sugar beets with an enhanced field emergence potential.

The availability of the complete genome sequence of the model plant *Arabidopsis thaliana*, together with the development of high-throughput procedures for global analysis of gene functions has launched the 'post-genomic' era in plant biology [24,25]. To gain insight into the sugar beet germination we initiated an analysis of gene expression taking advantage of recently developed genomic tools. Here we present a collection of 2,784 Expressed Sequence Tags (ESTs) derived from dry mature and germinating sugar beet seeds and examine temporal transcriptional profiles of the corresponding genes during germination using dedicated cDNA macroarrays. mRNA profiles were investigated for seeds germinated under standard conditions of temperature and water supply as well as for seeds germinated under multistress conditions combining salt, osmotic, liquid excess and reduced temperature stresses. Based on the obtained transcription data the main metabolic pathways, active during germination, could be described. By comparing the mRNA profiles of seeds germinated under various conditions, we were able to detect stress induced alterations in gene expression. This analysis sheds light on main adaptation mechanisms used by germinating sugar beet seeds to withstand stress.

## Methods

### Plant material

**Figure 1:**
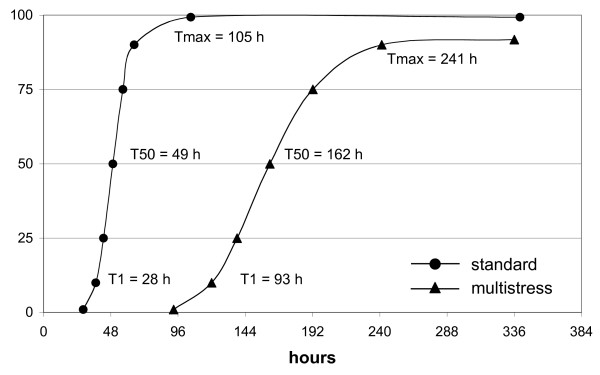
**Time-course of germination under standard and multistress conditions**. (●) – standard conditions: 30 ml of H_2_O, 15°C; (▲) – multistress conditions: 60 ml of 100 mM NaCl, 200 mM mannitol, 10°C. Data for each time point are averaged of three independent germination experiments. Germination parameters are evaluated by SeedCalculator 3.0 (Plant Research International, Wageningen).

Throughout the paper the term 'seed' refers to the sugar beet dispersal unit or fruit, which includes both the 'botanically true' seed and the pericarp. A good quality seed lot of a triploid monogerm sugar beet hybrid 302-688C (KWS SAAT AG) was used in this study. Seeds were produced in Italy in 2002 and processed (cleaned, polished, calibrated) after harvest according to KWS commercial standards. Germination experiments were carried out in three replicates each of 400 seeds in accordance with the International Seed Testing Association (ISTA) regulations. Germination parameters were evaluated by use of a special software: SeedCalculator 3.0 (Plant Research International, Wageningen). The seeds were incubated in plastic boxes (120 × 160 × 60 mm, 100 seeds per box) with pleated filter paper under two environmental conditions. The first, referred later to as 'standard', implies germination on pleated filter paper soaked with 30 ml of de-ionized water in the dark at 15°C. The second, referred to as 'multistress', characterizes germination on filter paper soaked with 60 ml of an osmotic solution (100 mM NaCl, 200 mM mannitol) in the dark at 10°C. Multistress conditions were defined after a set of pretrials evaluating the influence of single stress factors (different concentrations of NaCl, KCl and mannitol, excess of water, reduced temperature) as well as their combinations on germination characteristics (data not shown). The finally selected conditions caused a pronounced delay of germination but only a slight reduction in final germination percentage (Fig. 1). After evaluation of germination parameters samples corresponding to the beginning (T1, 1% of germinated seeds), progress T50, 50% of germinated seeds) and end of germination/seedling establishment (Tmax, nearly maximal % of germinated seeds) were obtained in larger quantities (3 biological repetitions, 700 seeds/seedlings in each sample). These samples, each represented by a mixture of non-germinated and germinated seeds at a certain proportion, were used for RNA isolation, cDNA library syntheses and transcriptome analyses.

### cDNA libraries

For RNA isolation 5 g of dry processed seeds (appr. 450 polished fruits) or germinating seeds at stages T1, T50 and T95 were homogenized in liquid nitrogen using a ball mixer mill MM200 (Retsch, Germany). Total RNA was isolated as previously described [26,27]. The average RNA yield varied from 2.5 mg for dry seeds (0.05% of starting material) to 8 mg for germinating seeds (0.16% of starting material, stage Tmax). Poly(A)^+^-RNA was extracted by Oligotex kit (Qiagen, Hilden, Germany). A cDNA library from dry seeds consisting of 21,000 clones was developed earlier (Windhövel and Westhoff, unpublished) from poly(A)^+^-RNA of 19 commercial sugar beet hybrids (KWS SAAT AG) mixed in equal proportions. This library was constructed using the SuperScript™ Plasmid System for cDNA Synthesis and Plasmid Cloning (Gibco BRL, Eggenstein, Germany). The synthesized cDNAs were inserted into the plasmid vector pSPORT1 predigested with *Not*I and *Sal*I restriction enzymes. Three cDNA libraries specific for different germination stages (T1, T50, Tmax), each consisting of 20,000 clones, were developed from seeds of the hybrid 302-688C germinated under standard conditions using the pBluescript II XR cDNA library construction kit (Stratagene, CA, USA). The derived cDNAs were ligated into the vector pBluescript II SK (+) predigested with *EcoR*I and *Xho*I restriction enzymes.

For cDNA library screening high-density filters containing 18,432 colonies per 22.5 × 22.5 cm^2 ^nylon membrane (Hybond N^+^, Amersham Bioscience) were produced. In order to avoid repetitive sequencing of identical clones, i.e. to reduce the library redundancy, normalization of the libraries was done using the following strategy. First 96 cDNA clones were randomly selected for sequencing. Then their inserts were PCR amplified and used as probes for colony hybridization with the high-density filters. Hybridizations were carried out with non-radioactive DIG-labeled probes according to the application manual of the manufacturer (Roche, Switzerland). Clones giving positive hybridization signals were sorted out and additional 96 cDNA clones were sequenced. The procedure of the subsequent sequencing and hybridization was repeated 19 times and at the end about 40% of the cloned cDNAs were found to be repetitive.

### Sequencing and bioinformatics

Expressed Sequence Tags (ESTs) were obtained by commercial sequencing of the selected cDNA clones (AGOWA, Berlin, Germany). cDNA clones derived from dry seeds were sequenced either from 3'-ends using the M13 forward primer (5'-CCAGGGTTTTCCCAGTCACG) or from 5'-ends using the T7 primer (5'-TAATACGACTCACTATAGGG). cDNA clones obtained from the germinating seeds were sequenced from 5'-ends using the M13 reverse primer (5'-CGGATAACAATTTCACACAGG). EST sequences are integrated at GabiPD database  and GenBank  and are accessible under the accession numbers [GenBank:FG343081-FG345864]. Sequences were subjected to clustering and assembly analysis with known sugar beet ESTs available at the GeneBank. Obtained consensus sequences were annotated against public nucleotide and protein databases and referred to different functional categories using Pedant-Pro™ Sequence Analysis Suite version 3 (Biomax Informatics AG, Martinsried, Germany). Additionally, Blast searches were performed against the current set of *Arabidopsis thaliana *proteins (TAIR database). Gene Ontology (GO) and TAGGIT [28] functional annotations of sugar beet sequences were done using the best *A. thaliana *hits detected with threshold levels of less than 1e-20.

To compare our data with a recently published set of sugar beet seed proteins [29] tBlastn searches of the ESTs against the available Mascot and Peaks peptides were carried out. A homology between an EST and a protein was considered to be reliable when it satisfied at least one of the following criteria: (1) one EST-peptide hit was detected with a minimum length of homology stretch of 10 amino acids (100% homology) or 15 amino acids (one mismatch, 93% homology); (3) at least two hits were detected between EST and different peptides corresponding to the same proteins with a minimum length of homology stretch of 7 amino acids (100% homology) or 11 amino acids (one mismatch, 90% homology).

### Preparation of cDNA macroarrays

PCR amplification was carried out from individual bacterial clones in 96-well plates using the primers 5'-GCAGGTACCGGTCCGGAATTCCCGGG and 5'-CCCAGTCACGACGTTGTAAAACGACGGCCA for cDNAs inserted into the vector pSPORT1 and the primers 5'-GTTGTAAAACGACGGCCAGTGAG and 5'-GCTATGACCATGATTACGCCAAGC for inserts ligated into the vector pBluescript II SK (+). The PCR program included an initial denaturation step, 3 min at 94°C, followed by 30 cycles of 20 sec at 94°C, 1 min at 60°C, 2 min at 72°C and a final extension step of 7 min at 72°C. Amplification of cDNAs isolated previously from sugar beet leaf, root and inflorescence libraries [28] and kindly provided by KWS SAAT AG was performed as described in Herwig et al. [28]. Amplification products were checked for purity and concentration by gel electrophoresis. High-density filter macroarrays representing 7.5 × 11.5 cm nylon membranes with double spotted PCR fragments of 1,047 cDNA clones from dry sugar beet seeds, 1,705 clones from germinating seeds and 216 cDNAs from sugar beet leaf, root and inflorescences [28] were developed commercially (RZPD, Berlin, Germany).

### Array hybridization

Array hybridizations were performed at least twice per each time point using different biological replicates of plant material. The synthesis of ^33^P-labeled cDNA was performed as described by Sreenivasulu et al. [30]. Hybridizations were carried out overnight at 65°C in Church buffer (250 mM sodium phosphate, pH 7.2, 7% (w/v) SDS, 1% (w/v) BSA, 1 mM EDTA). After hybridizations the membranes were washed for 20 min at 65°C in the subsequent solutions: 2 × SSC, 0.1% SDS; 1 × SSC, 0.1% SDS and 0.1 × SSC, 0.1% SDS. Then the membranes were wrapped in Saran film and exposed to an image plate of a Fluorescent Image Analyzer FLA-3000 (FujiFilm) for 12–24 hours. Labelled probes were stripped by placement of the membrane into boiling wash solution (0.1 × SSC, 0.1% SDS) and subsequent treatment in the solution for 30 min at 80°C. Successful removal of the radioactive probe was controlled by overnight exposure of the membrane.

### Data processing

The image data were evaluated by the AIDA program package (Raytest, Germany). Signal intensities were normalized with the total amount of radioactivity bound to the arrays. Empty spots were used for background calculation with the 'weighted background dots' function in the AIDA Image Analyzer software. The intensities of double spots representing the same cDNA were averaged. Macroarray hybridization quality tests made by comparison of technical replicates for the stages T0 and T50 revealed a high reproducibility of results (correlation coefficients r^2 ^= 0.99–1.00). Furthermore a high correlation between signal intensities representing biological replicates was observed (correlation coefficients r^2 ^= 0.97–0.99). It was noted that low-intensity spots demonstrated a greater variability than middle and high intensity spots. To reduce the number of false positives, the spots showing normalized intensities below four averaged background levels over all analyzed time-course stages were removed from further consideration.

Two criteria were applied for identification of differentially expressed genes. The first is a ratio-voting criterion based on more than 2-fold differences in averaged spot intensities and the second is a significance analysis of time course experiments based on Q-value evaluation [31]. The Q-value evaluation is convenient for producing a list of differentially expressed genes for any desired false discovery rate (FDR). Calculation of Q-values was carried out using the program EDGE [32]. Significance threshold q < 0.01 (1% FDR) was applied to the data. An EDGE input file contained 8 × 2,968 data points for 2,968 cDNA spots assayed over four time-course stages for two biological replicates. Hierarchical and k-mean clustering of differentially expressed genes was performed using Genesis 1.0 software [33]. Similarity distances were calculated based on Pearson's correlation coefficient. Hierarchical cluster analysis was done using the unweighted pair-group method with arithmetic averages (UPGMA).

### Northern and RNA dot blot hybridizations

For Northern analysis 10 μg of glyoxal denatured total RNAs isolated from dry and germinating sugar beet seeds were fractionated in 1% agarose gels and transferred onto Hybond N^+ ^membranes (Amersham Bioscience). For RNA dot blot analysis 5 μg of RNAs were loaded directly on membranes using a minifold dot-blot system (Schleicher&Schuell, Germany). The membranes were hybridized with ^32^P-labelled probes prepared according to Megaprime DNA Labeling System protocol (Amersham Bioscience). For RNA loading control the membranes were stripped and hybridized again with 18S rDNA probe.

## Results

### EST generation, clustering, annotation and comparison with proteome data

Sugar beet ESTs were obtained by single-pass partial sequencing of selected cDNA clones of four representative cDNA libraries developed from dry mature seeds and seeds germinated under standard conditions. In total 2,784 ESTs were generated including 1,170 from dry seeds and 1,614 from germinated seeds. To estimate the number of genes represented by the entire EST data set, the sequences were assembled into contigs. With 1,917 singletons and 334 contigs consisting of 871 ESTs, the calculated gene number was 2,251. The contigs contain 2 to 12 ESTs with the largest contig of 12 ESTs showing similarity to a late embryogenesis abundant (LEA) protein and the second largest one of 10 ESTs similar to the 60S ribosomal protein L41.

In order to obtain a better sequence annotation consensus sequences were generated for each contig. The consensus and singleton sequences making together the set of 2,251 'unigenes' were searched (BlastX) against a non-redundant protein database. As a result 1,670 sequences (74%) could be annotated to known or hypothetical plant proteins under the applied threshold value of 1e-20. In addition, the 'unigenes' were searched against the current set of *Arabidopsis thaliana *proteins available at 'The Arabidopsis Information Resource' . Gene ontology (GO) classification was done for 1,576 (70%) of sugar beet sequences using the best *A. thaliana *hits detected with threshold levels of less than 1e-20 (data not shown). Carrera et al. [34] reported that the ontological classification supplied by GO terms failed to provide much useful biological information in relation to seed biology. Therefore, the authors developed TAGGIT, a workflow that reannotated *A. thaliana *gene lists in relation to previously reported functions involved in embryo maturation, dormancy and germination. As only 21% of the *A. thaliana *hits comprising 14% of the total 'unigene' set could be annotated using the TAGGIT approach it was not further followed. Instead, sugar beet genes were manually annotated of using the combined information obtained by the different approaches.

Recently Catusse et al. [29] performed a proteome-wide characterization of sugar beet seed enabling the identification of 759 seed-specific proteins. As this analysis was done with the same sugar beet line 302-688C that was used in our study we have compared available transcriptome and proteome data. Searches for homology (tBlastn) between 2,784 sugar beet ESTs and peptide sequences [29] revealed that more than half of the proteins (453/759, 59.7%) could be attributed to specific ESTs. Conversely, just around 13.5% of the ESTs showed significant homology to the peptides/proteins (data not shown). This observation could be explained by the fact that in many cases one EST matched to several protein spots with identical annotation.

### Gene expression profiling of germination under standard conditions

#### Macroarray data analysis and overall expression patterns

Under standard conditions (30 ml H_2_O, 15°C) sugar beet seeds started to germinate after one day of imbibition (T1 = 28 h, 1% of germinated seeds), passed the time point T50 corresponding to 50% of germinated seeds at day 2 (T50 = 49 h) and reached a maximum germination value after day 4 (Tmax = 105 h) (Fig. 1). For simplicity, throughout the paper we refer to the analysed time points T1, T50 and Tmax as different 'germination stages' (beginning, progress and end of germination, respectively) though keeping in mind that Tmax can be also referred as 'seedling establishment' since at this time point the seeds have left the germination processes already. Gene expression profiles of dry mature sugar beet seeds (T0) as well as of the three germination stages were analysed in this study.

Macroarrays used for transcriptome analysis contained 2,752 cDNA fragments representing the whole set of 2,251 'unigenes'. In addition 216 cDNAs derived from sugar beet leaf, root and inflorescences [28] were included. They had been selected based on sequences homology to the published genes and/or proteins known to be expressed during germination or after induction by stress [7,20,35,36]. Because our macroarray quality test showed a high correlation between technical replicates, two biological replicates per each time point were examined. Of 2,968 cDNA spots represented on the macroarray, 2,056 (69%) showed mean hybridization signals above the selected threshold value for at least one time point. The hybridization profiles of these 2,056 cDNAs corresponding to 1,602 genes were analysed further.

Reliability of the results was additively verified by evaluating the expression patterns of those cDNA spots that were assigned to contigs. Three hundred twenty three of the 334 contigs (97%) analysed revealed a high correlation of the expression profiles among the different contig members. The occurrence of two dissimilar transcription patterns observed in the remaining 11 contigs suggests an inefficient resolution of genes representing gene family members. It should be noted that contigs were constructed based on partial, mostly 5'-end, sequences of the genes. Previously, it was shown that 5' sequence information is superior regarding gene discovery while sequence information obtained from the 3'-end is more useful for separation of individual members of gene families [37]. Thus, the data illustrate the importance and necessity of additional 3'-end sequence data for a successful gene family dissection.

**Figure 2:**
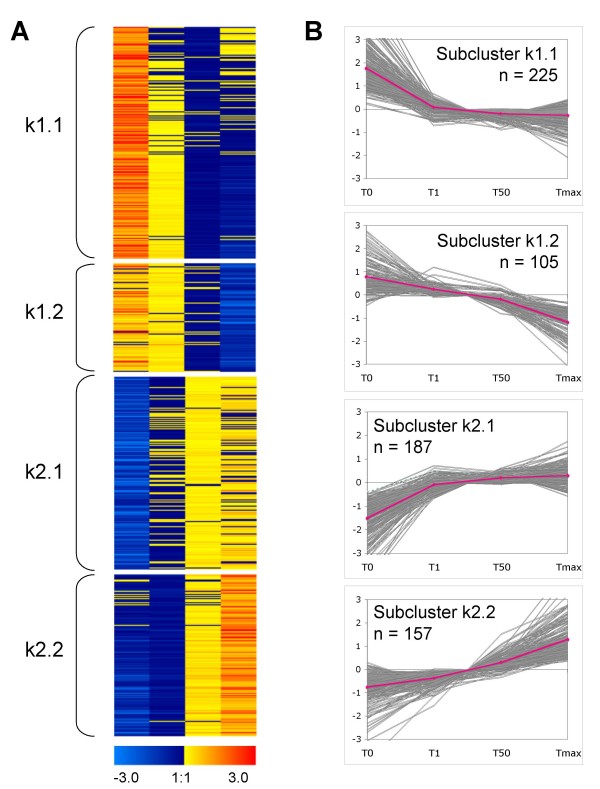
**K-mean clustering of 674 genes expressed differentially during germination under standard conditions**. Gene expression was monitored in dry (T0) and germinating (T1, T50, Tmax) seeds. Transcript abundance is shown as Log_2_-transform and gene-centered signal intensities. (A) Every horizontal row represents an individual gene. Red indicates transcript abundance above the mean and blue indicates transcript abundance below the mean (see also colour scale at bottom). (B) Temporal alterations of transcript abundance are shown. Transcript profile of each gene is shown as a grey line, magenta lines indicate the averaged expression values for each cluster.

**Figure 3:**
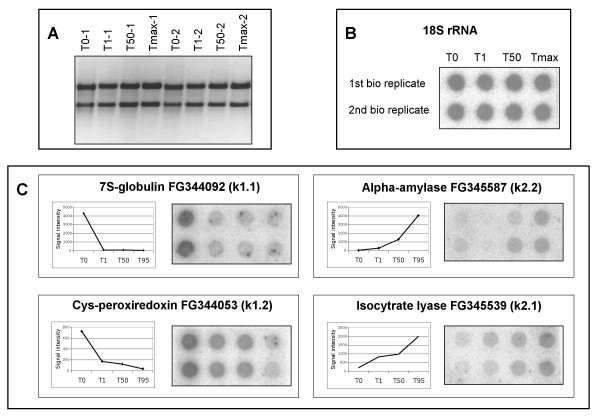
**Comparison of results obtained by macroarray and RNA dot-blot analysis**. (A) Gel-electrophoresis of RNA samples used for experiments, 1 μg of total RNA is loaded. (B) Control RNA dot-blot: 18S rRNA. (C) Graphical representations of gene expression data obtained by macroarray technique are shown adjacent to the corresponding RNA dot-blots. For (B) and (C): 5 μg of total RNA is loaded on a membrane and hybridized with a ^32^P-labelled probe.

Out of the 1,602 genes analysed, 1,088 (67.9%) showed at least a 2-fold change in transcript level in the course of germination, 361 genes (22.5%) revealed a more than 5-fold change and 202 genes (12.6%) a more than 10-fold change. A given gene was declared to be differentially expressed when it satisfied two criteria: (1) at least a 2-fold change in mRNA level during germination and (2) statistical significance under the applied threshold cut-off q < 0.01 (1% FDR) [31,32]. The final list of differentially expressed genes comprises 674 transcripts (Additional file 1). Most of the genes showed a gradual up- or down-regulation during the time course, and the most prominent changes in gene expression were observed between dry seeds (T0) and imbibed seeds (T1). A hierarchical and k-mean clustering divided the genes into two big clusters containing 330 down- and 344 upregulated transcripts. Based on the kinetics of gene expression each cluster could be further partitioned into two subclusters (Fig. 2). The down-regulated subcluster k1.1 and the up-regulated subcluster k2.1 are characterized by pronounced changes of gene expression in the beginning of germination followed by a plateau, while the subclusters k1.2 and k2.2 had more steady time-dependent kinetic patterns. Expression patterns of several genes from each subcluster were validated by RNA dot-blot and northern blot hybridizations and representative results are shown in Figure 3.

For 266 transcripts of cluster k1 and 301 transcripts of cluster k2 sequence analyses retrieved annotations with expected values below 1e-20. To compare the clusters of down- and upregulated genes the differentially regulated transcripts were assigned to different functional categories (Table 1). Three categories were highly overrepresented among down-regulated genes: (1) transcription, including nine genes showing similarity to known or putative transcription factors (TFs), (2) RNA metabolism, comprised mostly of pre-mRNA splicing factors and RNA-helicases, and (3) seed specific proteins including seed storage proteins (SSPs), late embryogenesis abundant (LEA), seed maturation and dehydration proteins. The most striking feature of the upregulated cluster k2 is a burst in a number of transcripts associated with energy production. Several functional subcategories, being absent in the cluster of downregulated genes, appear among the upregulated genes, i.e. mitochondrial energy conversion, tricarboxylic acid cycle (TCA), pentose phosphate pathway, photorespiration, glyoxylate cycle and gluconeogenesis (Table 1). Furthermore, the cluster is enriched in genes associated with photosynthesis, glycolysis, protein folding, carbohydrate, lipid and amino acid metabolism, cell wall metabolism, metabolism of various compounds, cell cycle/DNA processing and stress response.

**Table 1:** Functional classification of genes expressed differentially during germination under standard conditions.

**Functional category**	**Cluster k1**			**Cluster k2**		
	k1.1	k1.2	**Total**	k2.1	k2.2	**Total**
**Transcription**	11	5	**16**	-	-	**-**
**Seed specific proteins**						
Storage proteins	6	6	**12**	-	2	**2**
Maturation proteins	10	10	**20**	-	-	**-**
**RNA metabolism**	8	4	**12**	2	-	**2**
**Protein metabolism**						
Protein biosynthesis	13	16	**29**	17	9	**26**
Post-translation modification and folding	17	1	**18**	17	13	**30**
Protein catabolism	12	4	**16**	8	4	**12**
**Amino acid metabolism**						
Methionine cycle	-	-	**-**	3	1	**4**
Other amino acid metabolism	2	-	**2**	2	-	**2**
**Lipid metabolism**						
Lipid biosynthesis	1	-	**1**	-	1	**1**
Lipid degradation	1	-	**1**	4	-	**4**
**Carbohydrate metabolism**						
Starch, sucrose biosynthesis	-	-	**-**	-	2	**2**
Starch degradation	-	-	**-**	1	1	**2**
Other	-	1	**1**	2	2	**4**
**Secondary metabolism**						
Cell wall biosynthesis	-	-	**-**	3	3	**6**
Cell wall loosening	-	-	**-**	2	4	**6**
**Metabolism of various compounds**	7	2	**9**	16	8	**24**
**Energy**						
Glycolysis	1	-	**1**	3	7	**10**
Gluconeogenesis	-	-	**-**	-	1	**1**
Glyoxylate cycle	-	-	**-**	1	-	**1**
Pentose phosphate pathway	-	-	**-**	2	5	**7**
Tricarboxylic acid cycle	-	-	**-**	4	1	**5**
Photorespiration	-	-	**-**	4	2	**6**
Photosynthesis	1	-	**1**	1	6	**7**
Mitochondrial energy conversion	-	-	**-**	8	2	**10**
**Phytohormone regulation**	1	-	**1**	1	3	**4**
**Transport**	16	5	**21**	15	13	**28**
**Signal transduction**	8	4	**12**	4	4	**8**
**Response to stress**	9	1	**10**	8	11	**19**
**Cell cycle and DNA processing**	2	-	**2**	15	3	**18**
**Unclassified**	11	2	**13**	2	2	**4**
**Unidentified function**	44	24	**68**	18	28	**46**
**Not significant homology (> 1e-20)**	44	20	**64**	24	19	**43**

**Total**	225	105	**330**	187	157	**344**

#### Characterization of abundant mRNA species

**Figure 4:**
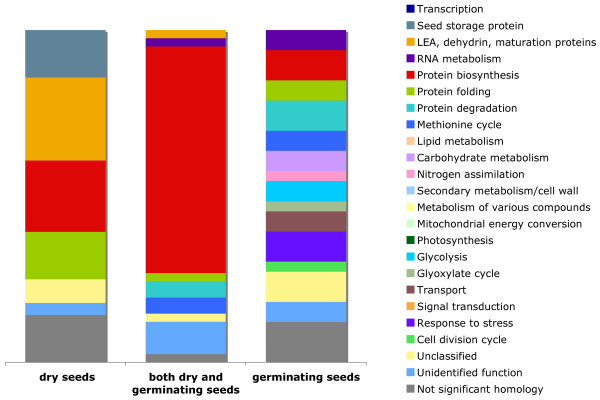
**Functional classification of abundant mRNA species**. Highly abundant transcripts are divided in three groups: preferentially expressed in dry seeds (28 genes), highly expressed both in dry and germinating seeds (41 genes) and preferentially expressed in germinating seeds (33 genes).

A total of 102 genes, showing averaged normalized signal intensities above 1,000 units at least once during the time-course, were considered to be highly expressed and were divided into three classes: abundant in dry seeds (28 genes), abundant both in dry and germinating seeds (41 genes) and abundant in germinating seeds (33 genes) (Fig. 4 and Additional file 2). The abundance of many proteins is regulated on the transcriptional level [38]. Accordingly, enhanced gene transcription might indicate increased biosynthesis of the encoded proteins and activation of the corresponding metabolic pathways. Indeed, the search for homology between the highly abundant transcripts and the sugar beet seed-specific peptides [29] results in more EST-protein hits (28/102, 27%) than have been found for the whole EST set (13.5%).

A prevailing number of transcripts abundant both in dry and germinating seeds (28 of 41) encode ribosomal proteins and eukaryotic elongation factors. This finding emphasizes a central role of protein biosynthesis during both seed maturation and germination and is consistent with previous studies [6,39]. Among other transcripts of this class are several genes encoding heat shock proteins (HSPs), chaperonins and ubiquitins associated with protein folding and catabolism.

As expected, dry mature seeds contain a large amount of mRNAs coding for SSPs and LEAs, the proteins predominantly synthesized prior to seed desiccation. Germinating seeds are characterized by a more diverse spectrum of abundant mRNA species (Fig. 4, Additional file 2). These include transcripts of several cysteine protease and α-amylase genes suggesting an active mobilization of protein and starch reserves during germination. Abundant transcripts of isocitrate lyase (ICL) (Additional file 2), the key enzyme of the glyoxylate cycle, imply the effective catabolism of the third storage compound, the lipids. The degradation of storage compounds might be associated with increased fluxes through the glycolytic pathway and active reassimilation of released ammonium as deduced from high mRNA levels of glyceraldehyde 3-phosphate dehydrogenase and glutamine synthetase genes, respectively. Germinating seeds were also characterized by abundant transcripts encoding superoxide dismutase, an enzyme scavenging reactive oxygen species (ROS).

Several genes encoding enzymes involved in methionine biosynthesis and the corresponding regeneration pathways, are found to be actively transcribed either in germinating (methionine synthase and adenosylhomocysteinase) or both in dry and germinating (S-adenosyl-L-methionine synthase) seeds. This finding is in agreement with a previous report on a high representation of ESTs related to one-carbon metabolism in 4-day old sugar beet seedlings [21]. Methionine synthesized by methionine synthase is not only used as a building block for protein synthesis but is also metabolized further into S-adenosyl-L-methionine (AdoMet), a well-known methyl donor in transmethylation reactions and substrate for ethylene, polyamines and biotin biosynthesis [40]. Recently, the importance of methionine and/or its derivatives for promoting germination and seedling growth has been demonstrated in *Arabidopsis *[41].

#### Mobilisation of seed storage reserves during germination of sugar beet seeds

Taking into account the important role of seed storage reserves for germination and early seedling vigour we focused on the analysis of genes that are involved in protein, starch and lipid degradation pathways in sugar beet. Given that the degradation of cellular proteins is a constant and ongoing process, the genes that are involved specifically in SSP degradation have to be identified first. Three vacuolar cysteine proteases (FG344171, FG345664 and FG345425), whose mRNA levels were low in dry seeds and upregulated 25 to 85 fold during germination, were assumed to be associated with SSP breakdown occurring inside protein storage vacuoles (Table 2).

**Table 2:** Specification of transcripts showing similarity to enzymes involved in starch and lipid reserve mobilization.^a^

**Reaction**	**Enzyme**	**GenBank accession number of EST showing similarity to the enzyme**	**Cluster**	**Maximal absolute expression under standard conditions**	**Relative expression under standard conditions**
**Starch degradation**					
**1**	Alpha-amylase (EC 3.2.1.1)	FG345587	k2.2	4240	148.2
**1**	Alpha-amylase (EC 3.2.1.1)	FG344655	k2.1	1820	43.1
**2**	Beta-amylase (EC 3.2.1.2)	FG345593	-	490	5.1
**2**	Beta-amylase (EC 3.2.1.2)	FG344086	-	205	0.3
**3**	Alpha-glucosidase (EC 3.2.1.20)	FG344078	-	5	1.0
**Glycolysis**					
**4**	Phosphoglucomutase (EC 5.4.2.2), cytosolic	BQ586122	-	70	1.0
**5**	UDP-glucose pyrophosphorylase (EC 2.7.7.9)	FG343121	-	110	3.2
**6**	Phosphofructokinase (PFK) (EC 2.7.1.11)	FG343255	k1.1	40	0.1
**7**	Pyrophosphate-fructose 6-phosphate 1-phosphotransferase (PFP) (EC 2.7.1.90)	FG345703	k2.2	180	2.7
**8**	Fructose-bisphosphate aldolase (EC 4.1.2.1), cytosolic	FG343104	-	510	2.7
**8**	Fructose-bisphosphate aldolase (EC 4.1.2.1), plastidic	FG345732	k2.2	180	10.4
**9**	Triosephosphate isomerase (TPI) (EC 5.3.1.1), cytosolic	FG344285	k2.2	400	7.9
**10**	Glyceraldehyde-3-P dehydrogenase (GAPDH) (EC 1.2.1.12), cytosolic	FG345028	k2.2	1980	2.3
**11**	Phosphoglycerate kinase (PGK) (EC2.7.2.3), cytosolic	FG343147	k2.2	575	3.8
**12**	Phosphoglycerate mutase (PGAM) (EC 5.4.2.1), cytosolic	BQ583729	-	35	2.4
**13**	Enolase (ENO) (EC 4.2.1.11), cytosolic	FG345118	k2.1	440	4.1
**14**	Pyruvate kinase (PK) (EC 2.7.1.40), cytosolic	FG344735	k2.1	210	2.1
**Pyruvate decarboxylation and fermentation**					
**15**	Pyruvate dehydrogenase E1 alpha subunit (EC 1.2.4.1)	FG345415	k2.1	80	4.9
**16**	Alcohol dehydrogenase (EC 1.1.1.1)	FG345166	k2.2	30	3.0
**16**	Alcohol dehydrogenase (EC 1.1.1.1)	FG343474	-	460	0.5
**Fatty acid beta-oxidation**					
**17**	Acyl-CoA synthetase (EC 6.2.1.3)	FG344059	-	165	3.5
**18**	Acyl-CoA oxidase ACX3 (EC 1.3.3.6)	FG345045	-	120	1.5
**19a**	Enoyl-CoA hydratase (EC 4.2.1.17)	FG345194	k2.1	40	5.6
**19b**	Multifunctional protein (EC 4.2.1.17, EC 1.1.1.35)	FG344653	-	625	1.3
**20**	Thiolase (EC 2.3.1.9)	FG344716	k2.1	295	10.4
**Glyoxylate cycle**					
**21**	ATP citrate synthase (EC 2.3.3.8)	FG345121	-	80	6.6
**22**	Aconitate hydratase (EC 4.2.1.3)	FG345009	k2.2	480	4.6
**23**	Isocitrate lyase, glyoxysomal (EC 4.1.3.1)	FG345539	k2.1	1970	9.4
**24**	Malate synthase, glyoxysomal (EC 2.3.3.9)	FG344826	-	690	2.5
**25**	Malate dehydrogenase (EC 1.1.1.37)	FG345685	k2.2	575	2.6
**Gluconeogenesis**					
**26**	Phosphoenolpyruvate carboxykinase (EC 4.1.1.49)	FG343814	k2.2	475	15.3
**Protein catabolism**					
**-**	Cysteine proteinase (EC 3.4.22.-)	FG344171	k2.1	1880	25.5
**-**	Cysteine-type endopeptidase (EC 3.4.22.1)	FG345664	k2.1	1700	85.0
**-**	Cysteine proteinase (EC 3.4.22.-)	FG345425	k2.2	505	50.5

^a ^Enzymes and the corresponding transcripts are arranged according the metabolic scheme shown in Figure 5. Relative expression indicates maximal fold of increment during germination.

**Figure 5:**
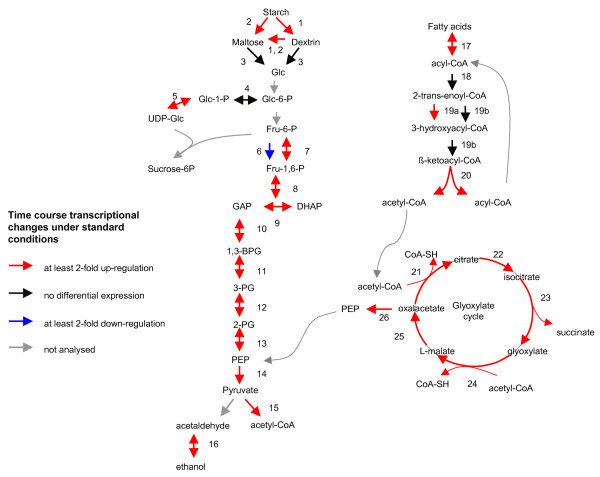
**Temporal transcriptional changes of genes involved in starch and lipid mobilization pathways during germination under standard conditions**. Enzymes designated by numbers are listed in Table 2. Transcriptional changes of the corresponding genes are depicted as coloured arrows with red indicating at least 2-fold up-regulation and blue indicating at least 2-fold down-regulation.

As deduced from EST analysis, starch in sugar beet seeds is degraded predominantly through the hydrolytic pathway (Table 2, Fig. 5 reactions 1–3). One of the analysed α-amylase genes (FG345587), with a low transcript level in dry seeds, showed an enormous upregulation (almost 150-fold) during germination and revealed the highest absolute mRNA level (4,240 units) among all the genes involved in reserve mobilization. Different time-dependent expression patterns were observed for two β-amylase genes, both characterised by medium mRNA level in dry seeds. One gene was 5-fold upregulated during germination whereas the second one was 3-fold downregulated. Amylase activity produces maltose, which is subsequently converted to glucose by the enzyme α-glucosidase. In contrast to medium to highly expressed amylase genes, the only gene with homology to α-glucosidase revealed a low mRNA level both in dry and germinating seeds. Given that alpha-glucosidases are encoded by several genes belonging to a large glycoside hydrolase gene family, we suppose that the specific family members involved in starch catabolism during germination were not represented on our array.

A complete set of ESTs encoding lipid degradation enzymes, including fatty acid β-oxidation and glyoxylate cycle, was detected in this study (Table 2, Fig. 5 reactions 17–25). The majority of genes identified had a medium mRNA level in dry seeds, indicating a possible flux through the pathways during seed maturation. Upregulation of most β-oxidation genes and all genes of the glyoxylate cycle (up to 10 fold) during germination justifies an increased flux through these pathways in germinating seeds.

The germination was accompanied by an increased transcription of genes that encode glycolytic enzymes (Table 2, Fig. 5 reactions 6–14). A coordinated upregulation was observed for all of these genes except for the ATP-dependent phosphofructokinase (PFK) that converts fructose-6-phosphate to fructose-1,6-bisphosphate. The down-regulation of the PFK gene and the opposite up-regulation of pyrophosphate-dependent phosphofructokinase (PFP) gene encoding an enzyme that catalyze the same but reversible reaction suggest a major role of PFP in maintenance of the flux through the glycolytic pathway during germination. Because all other glycolytic reactions are also reversible, the flux through the pathway can be directed either into the catabolism of glucose derived from starch or into the biosynthesis of glucose from metabolites derived from lipid degradation, glyoxylate cycle and gluconeogenesis. The upregulation of the transcript encoding phosphoenolpyruvate carboxykinase, the main enzyme of gluconeogenesis, during germination suggests an active reverse flux through the pathway.

### Alterations of gene expression during germination under multistress conditions

**Figure 6:**
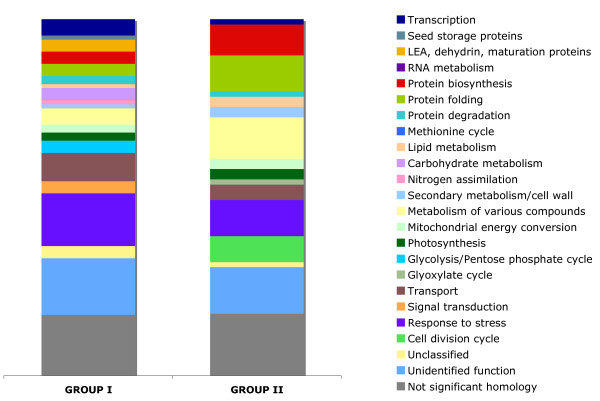
**Functional classification of stress-responsive genes**. Group I comprises 88 genes showing enhanced mRNA levels under stress conditions, group II consist of 69 genes showing reduced mRNA levels under stress conditions.

Multistress conditions combining salt (100 mM NaCl), osmotic (200 mM mannitol) and liquid excess (60 ml) stresses applied at a reduced temperature (10°C) delayed germination of sugar beet seeds significantly (162 hours to reach T50, 241 hours to Tmax) and decreased the maximum germination rate from 99% to 92% compared to the standard conditions (Fig. 1). Time-course expression profiling of 'multistress' germination was conducted according to the profiling of the 'standard' germination at three time points (T1, T50 and Tmax). To gain insight into the processes undergoing changes during 'multistress' compared to 'standard' germination we searched for genes expressed differentially in the two time-courses. Pairwise comparison of mRNA amounts at physiologically identical germination stages (T1, T50 and Tmax) revealed 157 genes showing at least 2-fold differences in expression levels (P < 0.05). Among these genes 88 demonstrated increased and 69 decreased mRNA levels in response to stress (Additional file 3). The first group of genes is characterized by an increased number of transcripts assigned to carbohydrate metabolism, glycolysis/pentose phosphate cycle, seed specific proteins, transport, response to stress, transcription, signal transduction, while the second group of genes demonstrates an enhanced number of transcripts involved in lipid metabolism/glyoxylate cycle, protein biosynthesis, protein folding, metabolism of various compounds and cell division cycle (Fig. 6).

**Figure 7:**
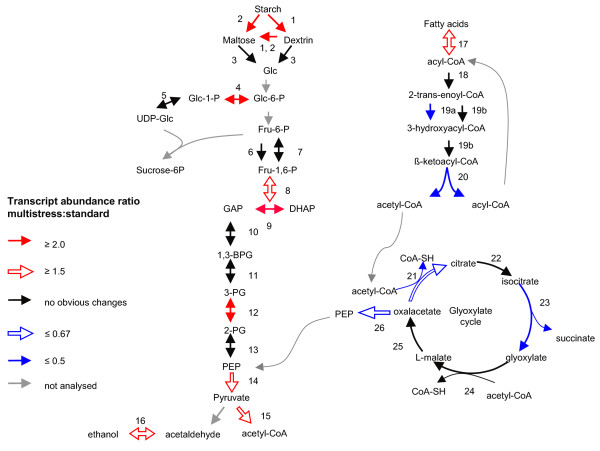
**Stress-induced transcriptional changes of genes involved in starch and lipid mobilization pathways**. Enzymes designated by numbers are listed in Table 2 and 3. Transcriptional changes are presented as maximal transcript abundance ratios at physiologically identical germination stages (multistress conditions to standard conditions). Red filled or opened arrows indicate enhanced transcript abundance (at least 2-fold or 1.5-fold) under multistress conditions and blue filled or opened arrows indicate reduced transcript abundance (at least 2-fold or 1.5-fold) under multistress conditions.

The striking finding is an altered expression of genes related to catabolism of starch and lipid storage reserves. Under the multistress conditions a stronger induction of genes involved in hydrolytic starch degradation (α- and β-amylases) and a weaker up-regulation of genes involved in lipid degradation (enoyl-CoA hydratase, thiolase and ICL) were observed (Table 3, Fig. 7). The enhanced amount of transcripts encoding starch-degrading enzymes coincides with a significant increase of mRNA levels of several genes attributed to glycolysis.

**Table 3:** Stress-induced transcriptional changes of genes involved in starch and lipid mobilization pathways.^a^

**Reaction**	**Enzyme**	**GenBank accession number of EST showing similarity to the enzyme**	**Maximal ratio of transcript abundance (multistress:standard)**	**Time point of maximal transcriptional differences**
**Starch degradation**				
**1**	Alpha-amylase (EC 3.2.1.1)	FG345587	3.1**	T1
**1**	Alpha-amylase (EC 3.2.1.1)	FG344655	1.9*	T1
**2**	Beta-amylase (EC 3.2.1.2)	FG345593	3.0*	T1
**Glycolysis**				
**4**	Phosphoglucomutase (EC 5.4.2.2), cytosolic	BQ586122	2.7**	T50
**8**	Fructose-bisphosphate aldolase (EC 4.1.2.1), cytosolic	FG343104	1.5	Tmax
**8**	Fructose-bisphosphate aldolase (EC 4.1.2.1), plastidic	FG345732	2.0*	T50
**9**	Triosephosphate isomerase (TPI) (EC 5.3.1.1), cytosolic	FG344285	2.2	T1
**12**	Phosphoglycerate mutase (PGAM) (EC 5.4.2.1), cytosolic	BQ583729	2.3*	T1
**14**	Pyruvate kinase (PK) (EC 2.7.1.40), cytosolic	FG344735	1.5	T1
**Pyruvate decarboxylation and fermentation**				
**15**	Pyruvate dehydrogenase E1 alpha subunit (EC 1.2.4.1)	FG345415	1.8*	T1
**16**	Alcohol dehydrogenase (EC 1.1.1.1)	FG343474	1.5	T1
**Fatty acid beta-oxydation**				
**17**	Acyl-CoA synthetase (EC 6.2.1.3)	FG344059	1.9*	T50
**19a**	Enoyl-CoA hydratase (EC 4.2.1.17)	FG345194	0.4**	Tmax
**20**	Thiolase (EC 2.3.1.9)	FG344716	0.5***	Tmax
**Glyoxylate cycle**				
**21**	ATP citrate synthase (EC 2.3.3.8)	FG345121	0.7*	Tmax
**23**	Isocitrate lyase, glyoxysomal (EC 4.1.3.1)	FG345539	0.5**	Tmax
**Gluconeogenesis**				
**26**	Phosphoenolpyruvate carboxykinase (EC 4.1.1.49)	FG343814	0.5**	Tmax

^a ^The transcripts are arranged according the metabolic scheme shown in Figures 4 and 5.

* P ≤ 0.05; ** P ≤ 0.01; *** P ≤ 0.001

'Multistress' germination results in increased mRNA levels of several genes encoding seed specific proteins (oleosin-like, seed maturation and LEA proteins) and transporters suggesting an essential role of these genes in osmotic stress adaptation and protection. Our finding of an enhanced expression of a gene encoding choline monooxygenase (CMO) indicates the activation of an additional pathway maintaining the osmotic potential of the cell, the accumulation of the osmoprotectant glycine betaine (GlyBet). The activation of this pathway is also supported by the enhanced expression of a downstream gene encoding betaine aldehyde dehydrogenase (BADH) (1.7-fold).

Reduced mRNA levels of several genes encoding cytoplasmic ribosomal proteins, translation initiation factors, histones and tubulin under stress conditions point out a negative influence of stress on protein biosynthesis, cell division and growth and are in agreement with literature data [42].

As expected, a considerable number of genes assigned to the stress response category, and especially those encoding ROS scavenging enzymes, showed an altered expression kinetic during germination under stress conditions, although not all of the genes demonstrated enhanced transcript amounts. The observed alterations of gene expression patterns are triggered by different signal transduction pathways and are governed by a hierarchical network of TFs. Two genes showing similarities to known protein kinases and three genes encoding putative TFs reveal enhanced transcript levels under stress conditions (Additional file 3) and are probably involved in stress regulation and signalling in sugar beet seeds.

## Discussion

### A new set of Expressed Sequence Tags (ESTs) from dry mature and germinating sugar beet seeds

In the current study we generated 2,784 sugar beet ESTs representing 2,251 unigenes. Notably, a considerable proportion of the ESTs (1,170) is derived from a dry mature seed specific cDNA library. To our knowledge no ESTs isolated directly from dry mature sugar beet seeds have been reported so far, although some of the publicly available ESTs, especially derived from immature inflorescences [28], field-harvested seed stalks [McGrath, unpublished] and germinated seedlings [20,21] might represent genes expressed in seeds.

Due to the applied cDNA library normalization technique based on several repetitive cycles of sequencing and colony hybridization (see Methods), the proportion of unique ESTs in the set was enriched up to 82%. For comparison de los Reyes et al. [20] have reported the percentage of unique ESTs between 50.5% to 59.7% when subtracted and unsubtracted cDNA libraries from 4-day-old, solution-germinated sugar beet seedlings were sequenced. In order to estimate the number of novel sequences contributed to GenBank BlastN searches of unigene sequences against known sugar beet EST were done. At least 776 ESTs (28%) represent novel sequences.

### Reorganization of gene expression during germination

Germination is a key developmental stage in plant life cycle. Upon imbibition, the dry quiescent seed resumes metabolic activity, and this metabolic switch is accompanied by a huge shift in gene expression. In the current study transcriptome profiles of dry mature and germinating sugar beet seeds were analysed using dedicated cDNA macroarrays representing 2, 251 unigenes. Recently it has been shown that as many as 12,470 mRNA species were quantitatively detected in dry *Arabidopsis thaliana *seeds with a similar number of transcripts (14,395) detected in 24 h-imbibed seeds [6]. Therefore it is reasonable to assume that only a certain percentage of the transcripts stored in dry sugar beet seeds and expressed during germination was examined in our study. Taking into account that (1) rare transcripts are likely underrepresented in the EST dataset and (2) there are limitations in the identification of rare transcripts by the applied macroarray technique, characterization of the more highly expressed fraction of the transcriptome is presented here.

More than half of the analysed sugar beet genes revealed 2-fold changes of mRNA levels during germination. An assay of the genes demonstrates a considerable overrepresentation of transcripts encoding SSPs, LEAs, TFs, pre-mRNA splicing factors and RNA helicases in the cluster of downregulated genes. SSP genes (globulins, vicilins and oleosins) are known to be strongly expressed during seed maturation and reserve accumulation while LEA genes become activated later and play a protective role during seed desiccation [24,43]. Both SSP and LEA genes are expected to be downregulated during seed germination coinciding with the degradation of the corresponding proteins. An increased amount of pre-mRNA splicing factors and RNA-helicases in dry seed suggests an important role of the corresponding proteins during seed maturation or/and desiccation. Recently, it has been proposed that RNA helicases may function as RNA chaperones by active disruption of misfolded RNA structures so that correct folding can occur [44]. It is tempting to speculate that RNA chaperones are involved in the protection of long-lived mRNAs, capable to survive seed desiccation, by forming specific ribonucleoprotein complexes.

Nine putative TFs belonging to six TF families (CCCH-type zinc finger, AP2-EREBP, bZIP, MYB, EIL and CCAAT-HAP3 box binding) were found to be downregulated during germination. Members of these TF families were implicated in controlling many aspects of seed maturation, such as accumulation of storage compounds, cotyledon identity, acquisition of desiccation tolerance, dormancy, seed mass and seed yield [45-48], in mediation of ABA response [49] and in the ethylene-signalling pathway [13,17]. Germination is commonly accompanied by an increased transcription of a number of TFs [13,36] but, surprisingly, no upregulated TFs were detected in our study. Among the possible explanations of this phenomenon are the already mentioned under-representation of rare transcripts on the macroarray as well as a transient character of transcriptome changes. The genome-wide profiling of germination in *A. thaliana *showed that the mRNA profile of the imbibed seeds differed from that of dry seeds already 6 h after seed imbibition [6]. Given that the first two stages of the performed time course (T0 and T1) were separated by a time period of 28 hours, rapid and transient mRNA changes occurring during early imbibition might remain undiscovered.

A high amount of the genes involved in energy production pathways, storage reserve mobilization, reinitiation of cell cycle, overall metabolism and activation of respiration was detected in the cluster of upregulated transcripts, thus confirming data reported for other species [3,6,9]. To address the question, whether functionally orthologous genes are transcribed in *A. thaliana *and *B. vulgaris*, the sugar beet transcripts detectable in dry (1,238) and germinating (1,340) seeds were annotated against *A. thaliana *genes and the obtained hits were compared with the mRNAs described by Nakabayashi et al. [6]. Most of the annotated sugar beet genes (93% for dry seeds and 94% for germinating seeds) were present among the transcripts detected in dry and 24 h-imbibed *A. thaliana *seeds, correspondingly. The high proportion of functionally orthologous genes transcribed in *Arabidopsis *and sugar beet seeds might reflect a general conservation of the mechanisms regulating seed maturation and germination in dicotyledonous species.

### Mobilisation of seed storage reserves during germination of sugar beet seeds

Amount and efficient mobilization of seed storage reserves are important parameters determining early seedling vigour and field emergence. Sugar beet seeds contain starch, proteins and lipids as storage reserves. A previous study of sugar beet germination and early growth has shown that lipids are the major metabolic substrate during germination *sensu stricto *and, with other seed reserves, are involved in the heterotrophic nutrition of young seedlings [1]. To examine, whether these data can be supported on the transcriptome level, we compared mRNA profiles of genes involved in starch, protein and lipid mobilization pathways. During germination the most pronounced upregulation of mRNA levels was observed for starch mobilizing enzymes (α-amylase, 148-fold), followed by protein degrading enzymes (cysteine protease, 85-fold) and, finally, enzymes involved in lipid degrading (ICL, 9.4-fold). Because of post-transcriptional regulation, different turnover rates and varying kinetic parameters, gene mRNA levels can be just limitedly used to predict the magnitude of enzyme activities. However, it is worth to mention that genes involved in the mobilization of storage compounds reveal similar high mRNA levels at the final stage of germination (Table 2). Remarkably, a weaker upregulation of ICL and other genes involved in lipid mobilization did not result in lower mRNA amounts at the end of germination because of initial higher mRNA levels in dry seeds. The latter might be linked to an earlier activation of the lipid mobilization pathway during germination.

The presented transcriptome data for seed storage reserve mobilization (Table 2, Fig. 5) are in excellent agreement with the results of proteome analysis of sugar beet seeds [29] where complete metabolic modules, such as protein degradation, starch metabolism, fatty acid beta-oxidation, glyoxylate cycle and glycolysis, have been identified. Nineteen out of 34 ESTs listed in Table 2 (56%) showed significant homology to peptide sequences published by Catusse et al. [29].

### Expression pattern analysis suggests enhanced carbohydrate and reduced lipid catabolism during germination under stress conditions

Drought or wetness, salt stress and non-optimal temperatures are critical environmental factors that limit seedling emergence in the field. Whereas some gene expression studies state that various stresses, like cold and drought, share a common set of signal transduction pathways [50], there are also reports showing that the majority of changes are stimulus specific [51]. To identify a broad range of genes important for seed and seedling stress tolerance we combined salt, osmotic, excess of liquid and reduced temperature stresses together in one assay. This approach resulted in the identification of 157 stress-responsive genes. The observed proportion of stress-responsive genes (9.8% of 1,602 genes detectable on the array) is comparable with 6.1% (49/807) of differentially regulated transcript-derived fragments identified in 4-day old sugar beet seedlings germinated on filter paper, in NaCl, mannitol and H_2_O_2 _solutions or in water [21].

We found that genes related to storage reserve catabolism are specifically affected by stress conditions during sugar beet germination: the mRNA levels of genes involved in starch degradation were increased, whereas the mRNA levels of genes related to lipid catabolism were decreased (Table 3, Fig. 7). Interestingly, these alterations coincide with an enhanced expression of several genes involved in glycolysis and a reduction (1.8-fold) in the expression of PEP carboxylase – an enzyme involved in gluconeogenesis. Taken together the data suggest that in the analysed sugar beet hybrid stress conditions cause a shift in the ability of the seed to metabolize seed storage compounds with a favoured degradation of starch in compensation for lipids.

Given that the stress germination was conducted on pleated filter paper moistened with 60 ml of a NaCl/mannitol solution instead of 30 ml of deionized water, the observed gene expression changes might be related to hypoxia generated by an excess of liquid functioning as a diffusion barrier for oxygen. Indeed, some of the observed mRNA alterations point out changes characteristic for oxygen deprivation. It is known that under anaerobic conditions, the rate of glucose metabolism increases accompanied by enhanced fermentation. Similarly, in our study an increased expression of several glycolytic genes and a slight enhancement in the amount of alcohol dehydrogenase transcripts (1.5-fold) were observed. In germinating but still heterotrophic seeds the growing demand for glucose can only be satisfied by an enhanced degradation of seed reserves. In hypoxic conditions starch degradation might be favoured compared to the mobilization of lipids because the fatty acid β-oxidation pathway requires molecular oxygen as electron acceptor.

Alterations in expression of genes involved in starch and lipid catabolism during sugar beet germination under stress conditions have been reported previously. De los Reyes et al. [20] examined germination of two sugar beet hybrids differing in seedling vigour. Stress imposed by germinating sugar beet seeds in solution remarkably reduced the number of α-amylase transcripts in the weakly emerging hybrid. In contrast, the strongly emerging hybrid exhibited only a moderate reduction in α-amylase mRNA level with a simultaneous increase in transcripts of genes encoding enzymes for lipid degradation, suggesting compensation by lipid for carbohydrate metabolism in the better emerging hybrid. Given that the sugar beet cultivar 302-688C analyzed in our study is known as a good emerging hybrid, the observed transcriptome changes are opposite to the ones described by de los Reyes et al. [20]. There are at least two possible reasons explaining this discrepancy: the different experimental setups (germination in 150 mM NaCl solution *versus *germination on pleated paper moistened with a 100 mM NaCl/200 mM mannitol solution) and/or the different genetic background of the analysed sugar beet hybrids. Apparently, the reaction of sugar beet to stress depends remarkably on genotype, and different hybrids may use distinct strategies to overcome stress injuries.

### Other transcriptome changes in response to stress conditions

Many genes respond to abiotic stresses at the transcriptional level, and these genes might be classified into two major groups. One group encodes products that directly protect plant cell against stresses (stress adaptation), whereas products of the other group regulate gene expression and signal transduction in stress responses (stress regulation) [52]. Genes belonging to both groups were detected in our study. The first group includes transcripts related to stress response and osmotic stress adaptation, e.g. ROS detoxifying enzymes, LEA and transporters, while the second group includes several TFs and kinases (Additional file 3).

The majority of the transcriptional changes are in agreement with data published previously. Thus, the increased amount of transcripts encoding ROS detoxifying enzymes have been widely described in the literature as being important for stress tolerance [23,35]. LEA proteins are known to accumulate in response to dehydration, low temperature, salinity or exogenous ABA treatment [43,53]. There are also evidences of an enhanced expression of some oleosin genes in response to dehydration, high salinity and ABA [54,55].

Accumulation of LEA, seed maturation and oleosin transcripts proposes that a stress germination results in an increased transcription of genes that are normally accumulated during seed maturation and are under ABA control. Since ABA is a potent inhibitor of seed germination it would also account for the delay in sugar beet germination observed under multistress conditions. Similar results have been reported by Rajjou et al. [23] who detected the reinduction of the late maturation program during early stages of germination in *A. thaliana *under stress caused by salicylic acid. These data are also in agreement with an existence of a small developmental window during which the seed can still recruit late maturation programs [56,57].

The enhanced expression of transporter genes in response to osmotic stress has been found for different plant species and reflects a necessary readjustment of cell water balance [43]. Accumulation of compatible solutes in the cytoplasm, including amino acids, ammonium compounds and polyols/sugars, lowers the osmotic potential of the cell without inferring with metabolic processes or protein structuring and functioning, and consequently, maintains the water content of the cell under stresses [58]. Recently the ability of sugar beet seeds to synthesize the osmoprotectant GlyBet has been demonstrated [29]. GlyBet is synthesized in chloroplasts through the two-step oxidation of choline catalysed by the two enzymes, CMO and BADH. Increased mRNA levels of both genes during stress germination suggest an enhanced biosynthesis of GlyBet in response to stress. This finding is in agreement with the previously observed accumulation of CMO and BADH mRNAs in sugar beet leaves and roots in response to salinity and drought [59,60], and proves the uniformity of the stress adaptation mechanism in sugar beet adult plants and seedlings.

At least three putative TFs belonging to AP2-EREBP, MYB and CCCH-type zinc finger families of TFs show enhanced gene expression during germination of sugar beet seeds under multistress conditions (Additional file 3). Whereas an involvement of MYB and AP2-EREBP TF families in ABA-dependent stress regulatory network is well known [52], an association of CCCH-type zinc finger TF family with stress response has not yet been observed in plants. Two genes related to signal transduction, serine-threonine protein kinase and sucrose non-fermenting-related protein kinase regulatory subunit, show increased mRNA amounts during germination under stress conditions. The sucrose non-fermenting-related kinase complex (SnRK1) of plants is a global regulator of carbon metabolism and is considered to be a crucial element of the transcriptional, metabolic and developmental regulation in response to stress [61,62]. Recent expression analysis of pea seeds with repressed SnRK1 activity proposed that the SnRK1 is a mediator of ABA function [63]. This finding strengthens our hypothesis on the induction of an ABA-dependent signalling pathway during sugar beet stress germination.

The conducted analysis of sugar beet stress-responsive transcripts revealed many similarities with other plant species. However, the biochemical function of at least 50 differentially expressed genes remains unknown and needs further investigation. A better characterization of stress-responsive genes and the mechanisms involved is of great economical importance since it opens perspectives to improve the seed and seedling stress resistance and, consequently, field emergence. An increasing number of reports demonstrate that TFs [64], ROS detoxifying enzymes [65], transporters [66], protein kinases [67], osmolyte synthesizing enzymes [68], LEAs [69] and dehydrins [70] can be successfully used for genetic engineering of plant stress tolerance. In the current study a substantial number of genes that belong to these functional categories has been detected. The major challenge ahead is to elucidate the relative contribution of each gene to sugar beet stress tolerance and field emergence, and to select candidates for trait improvement either via biotechnological or standard breeding approaches.

## Conclusion

The new developed EST collection from sugar beet seeds and seedlings overlaps substantially with a recently published set of sugar beet seed-specific proteins and provides a useful resource to investigate the molecular bases of seed development, maturation and germination. The conducted gene expression profiling experiments enabled us to compare the transcriptional changes during sugar beet germination with the corresponding processes in the model species *A. thaliana*, and manifested a general conservation of the mechanisms regulating germination in dicotyledons. The complete representation and the abundance of transcripts involved in starch degradation, beta-oxidation, glyoxylate cycle and glycolysis pathways are found to be in an excellent agreement with recently published proteome data and reinforces the importance of seed reserve mobilization for efficient germination and early seedling growth. Transcript profiles of dry and germinating seeds imply that the sugar beet seed is well prepared to mobilize lipids very early during imbibition, with genes encoding starch-degrading enzymes (α-amylases) being activated later. Interestingly, the subsequent assay of germination under multistress conditions revealed significant alterations in the expression of genes involved in seed storage reserve mobilization, and suggested that in the analysed hybrid stress caused a transcriptionally regulated metabolic shift resulting in a favoured degradation of starch and reduced utilization of lipids. The approach also succeeded in the identification of other stress-responsive genes including those encoding TFs, protein kinases, LEA proteins, ROS detoxifying and GlyBet synthesizing enzymes, which have potential to contribute towards improvement of seed and seedling stress tolerance and, subsequently, field emergence potential in sugar beet.

## Authors' contributions

EP conducted RNA isolation, cDNA library development from germinating seeds, macroarray hybridizations, data analysis and drafted the manuscript. AW developed cDNA library from dry seeds, made preliminary gene expression experiments and provided several protocols. JM made germination assays, calculated germination parameters and prepared seed samples. AM provided bioinformatics support including contig assembling and consensus sequence annotation. UF and PW coordinated the project. All authors read and approved the final manuscript.

## Supplementary Material

Additional file 1 **Table A1: **List of genes expressed differentially during germination under standard conditions. Click here for file

Additional file 2 **Table A2: **List of genes expressed abundantly in dry and germinating sugar beet seeds. Click here for file

Additional file 3 **Table A3: **List of genes showing significantly different transcript levels during germination under stress conditions. Click here for file
